# Heterogeneity of a landscape influences size of home range in a North American cervid

**DOI:** 10.1038/s41598-018-32937-7

**Published:** 2018-10-02

**Authors:** W. David Walter, Tyler S. Evans, David Stainbrook, Bret D. Wallingford, Christopher S. Rosenberry, Duane R. Diefenbach

**Affiliations:** 1 U.S. Geological Survey, Pennsylvania Cooperative Fish and Wildlife Research Unit, The Pennsylvania State University, University Park, PA 16802 USA; 2 Pennsylvania Cooperative Fish and Wildlife Research Unit, The Pennsylvania State University, University Park, PA 16802 USA; 3 Pennsylvania Game Commission, Bureau of Wildlife Management, Harrisburg, PA 17110 USA; 4 Present Address: West Virginia Division of Natural Resources, French Creek, Elkins, WV 26218 USA; 5 Present Address: Massachusetts Division of Fisheries and Wildlife, Westborough, MA 01581 USA

## Abstract

In the northeastern United States, chronic wasting disease has recently been detected in white-tailed deer (*Odocoileus virginianus*) populations, and understanding the relationship between landscape configuration and home range may improve disease surveillance and containment efforts. The objectives of our study were to compare size of home range for deer occupying a continuum of forested landscapes and to investigate relationships between size of home range and measures of landscape configuration. We used a movement-based kernel density estimator to estimate home range at five spatial scales among deer across study areas. We developed 7 linear regression models that used measures of the configuration of the forested landscape to explain size of home range. We observed differences in size of home range between sexes among areas that differed based on landscape configuration. We documented size of home range changed with various metrics that identifying connectivity of forested patches. Generally, size of home range increased with an increasing proportion of homogenous forest. Our results suggest that deer in our region occupy a landscape at hierarchically-nested scales that is controlled by the connectivity of the forested landscape across local or broad geographical regions.

## Introduction

Use of Global Positioning System (GPS) technology for tracking wildlife has resulted in estimators of home range that account for large numbers of locations and animal-specific data (e.g., habitat use, duration between locations) that was not possible with traditional estimators^1–3^. Traditional estimators of home range used outer-boundary polygons, kernel densities, or relatively sparse data collected using very high frequency (VHF) telemetry that only estimated the utilization distribution with location-based parameters (i.e., no temporal component included). In comparison, the movement-based kernel density estimator has the advantage of incorporating temporal components and habitat-specific movement vectors available with GPS technology^4^. Movement-based kernel density estimators (hereafter referred to as MKDE) incorporate serially correlated locations, duration between locations, positional error of GPS technology, and habitat to provide estimates of home range that better account for movements that relate to the landscape^4^. Applied use of MKDE to estimate home range can provide more refined shapes and scales of home range for analysis of landscape configuration and complexity in mammals^1,5^.

Spatial processes, including spread of disease, are affected by the scale at which deer establish home range and this can be related to landscape^6–8^. Understanding how landscape heterogeneity influences space use by cervids can assist wildlife managers with alleviating issues that include forest regeneration, crop damage, and disease transmission^9–11^. For example, the spatial distribution of chronic wasting disease was related to features of the relatively open landscapes in the West and Midwest in mule deer (*Odocoileus hemionus*)^12–14^ but has only recently been investigated in a predominantly forested ecosystem for white-tailed deer (*Odocoileus virginianus*) infected with chronic wasting disease^8^. Furthermore, it is likely that the spatial distribution of chronic wasting disease throughout North America is related to both movements of infected species and landscapes that relate to these movements. Therefore, understanding the configuration and complexity of landscapes at which deer establish home range can provide a basis for disease surveillance and containment.

To address the spatial complexities of disease transmission, spatial processes can be influenced by scale of study area and demographic composition of the species studied and must be understood^7,15,16^. These spatial processes, however, are rarely assessed in predominately non-migratory species occupying restricted geographic range or with limited dispersal/migratory distances^17–20^. Studies assessing spatial scale have routinely documented the influence, or lack thereof, for landscape features (e.g., roads, rivers, and forest cover) or demographic composition of the population sampled (e.g., sex, age) to influence size of home range for various cervid species^7,21,22^. The foundation for previous assessments of spatial heterogeneity and how they relate to size of home range, however, is based on primarily VHF datasets utilizing arbitrarily-defined buffer sizes or estimators of home range that are no longer viable with GPS datasets^1,2^.

Understanding the relationship between use of heterogenous landscapes by cervids and spatial distribution of disease is related to the configuration and complexity of landscapes at which home ranges are established and can provide insights to address disease surveillance and containment. Because there has been no standardized method to assess this relationship, we propose the use of a relatively new estimator of home range on a species that exhibits various movement behaviors and seasonal shifts over a limited geographic range. Our specific objectives were to (1) compare size of home range between sexes and among study areas for white-tailed deer occupying a continuum of forested landscapes from fragmented to homogenous, (2) investigate relationships between size of home range and measures of landscape composition and configuration, and (3) determine differences in this relationship across spatial scales as determined from percentages of utilization distributions as opposed to arbitrarily-defined buffers or estimators.

## Study Area

**Figure 1:**
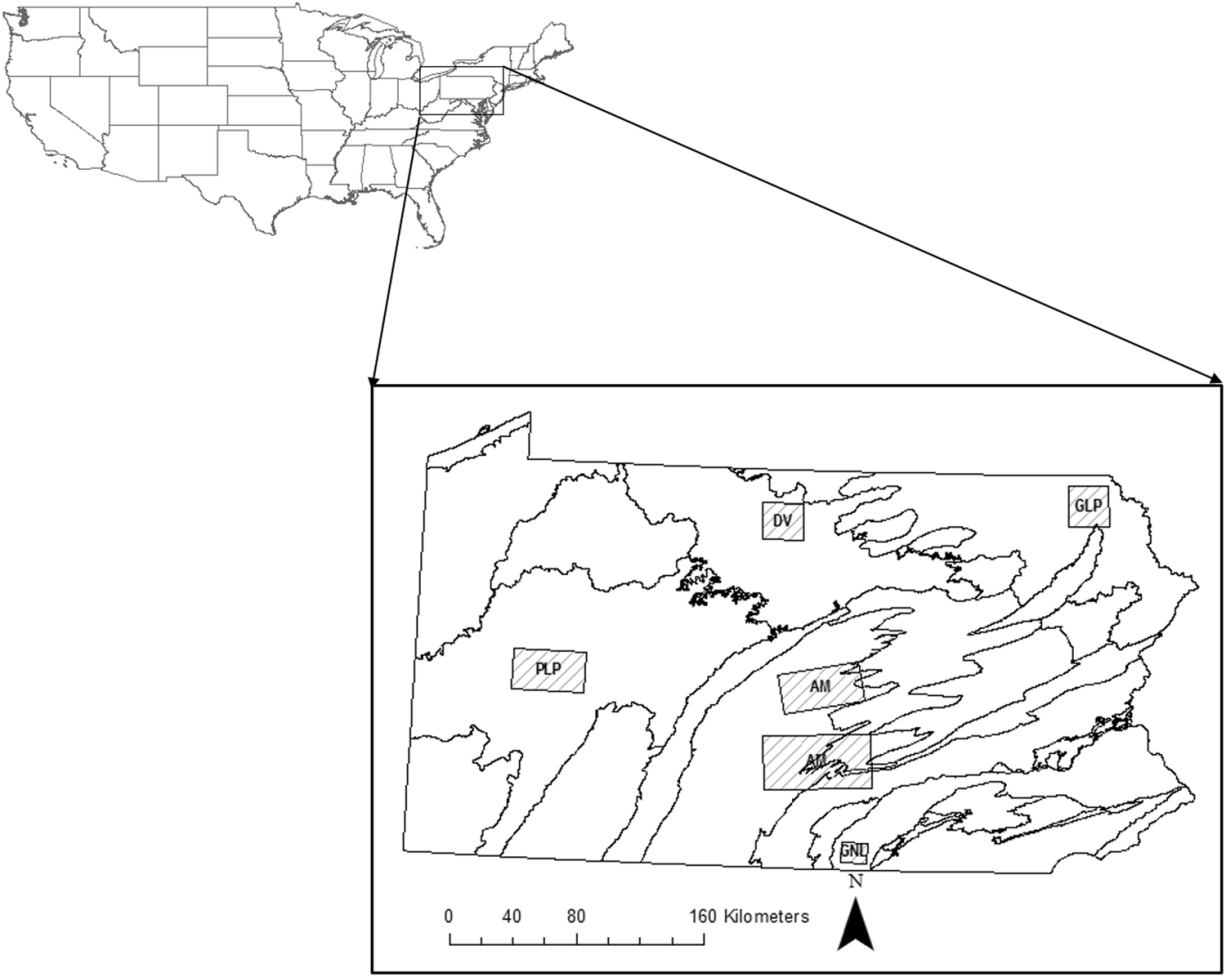
General location of study areas used to assess the relationship between heterogeneity of landscape and size of home range for white-tailed deer (*Odocoileus virginianus*) from 2009 to 2015 in Pennsylvania, USA. Cross stitched polygons represent physiographic province of study areas that included: Gettsyburg-Newark Lowland (GN), Pittburgh Low Plateau (PLP), Glaciated Low Plateau (GLP), Appalachian Mountains (AM), and Deep Valleys (DV). Generated with ArcMap 10.2, www.esri.com.

**Figure 2:**
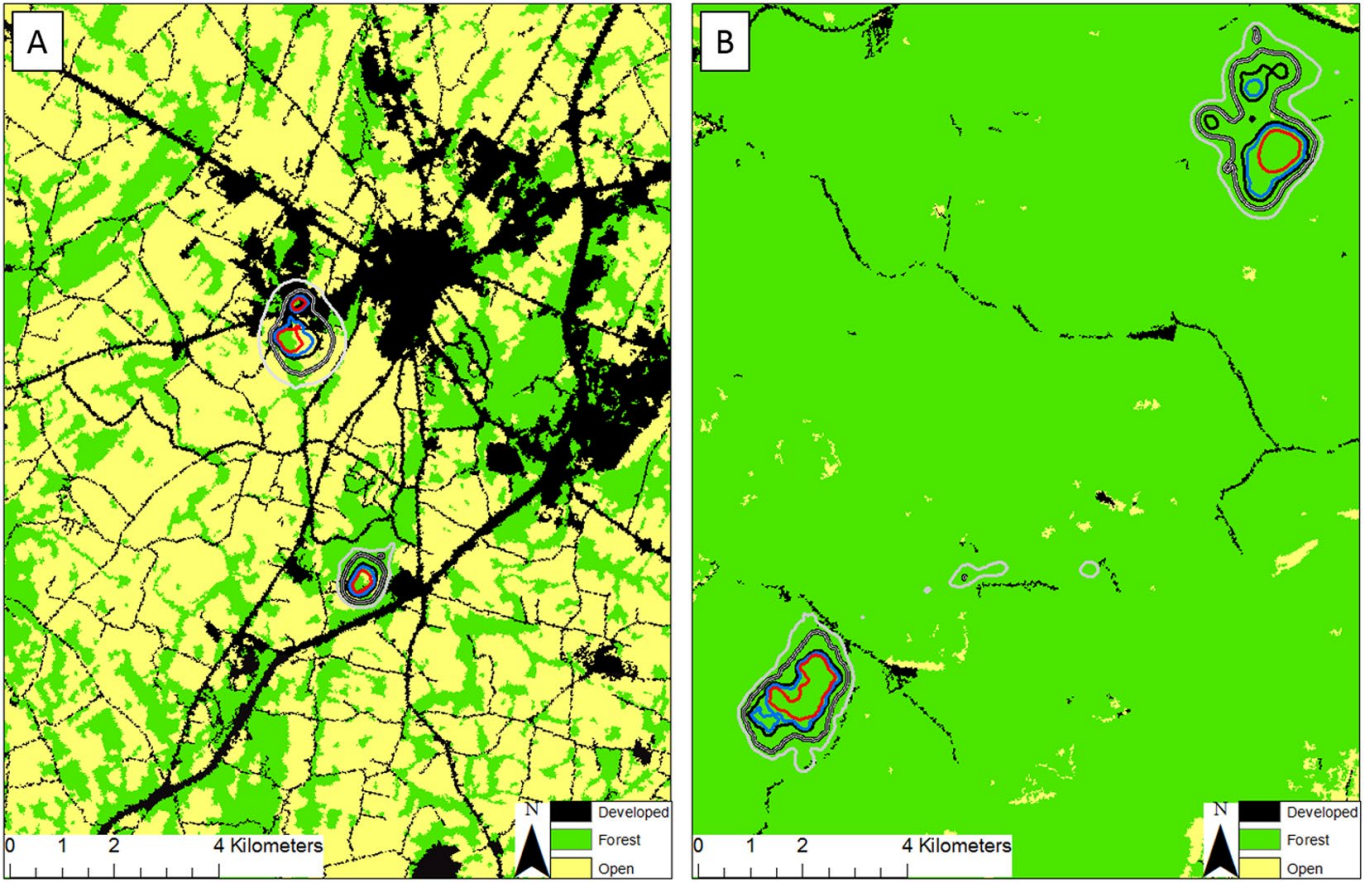
Habitat categories with isopleth polygons to assess the relationship between heterogeneity of landscape and size of home range for white-tailed deer (*Odocoileus virginianus*) from 2009 to 2015 in (**A**) Gettsyburg-Newark Lowland and (**B**) Deep Valleys in Pennsylvania, USA. Polygons reflects isopleths of home range at 50% (red), 70% (blue), 80% (black), 95% (black-gray), 99% (gray). Generated with ArcMap 10.2, www.esri.com.

We estimated size of home range for white-tailed deer in distinct geographical areas that represented a physiographic province in Pennsylvania, USA (Fig. 1)^23,24^. Each area represented a continuum of forested landscapes ranging from fragmented to homogeneous (Table 1; Fig. 2). Gettysburg-Newark Lowland was located in the Gettysburg National Military Park in central Adams County and elevation ranged from 87 m to 236 m. Pasture and cropland were dominant classes of land cover throughout the area with a largest patch size of 627 m^2^ (Table 1). Forest cover was sparse (22%) and fragmented in this area due to the dominant presence of anthropogenically-modified habitats. The Pittsburgh- and Glaciated-Low Plateaus were located in the western and northeastern region of the state, respectively (Table 1). These areas represented rural and moderately fragmented landscapes where open (e.g., pasture, cropland) and forested classes created a mosaic with a largest patch size of 929 m^2^ and elevation ranging from 225 m to 819 m (Table 1). The Appalachian Mountain study areas were located in central Pennsylvania and were characterized by contiguous forests along ridgelines and contiguous distributions of pasture and cropland in valleys with a largest patch size of 779 m^2^ (Table 1). Elevation in these areas ranged from 111 m to 737 m. The Deep Valley area was located in the north-central region of the state in homogeneous northern hardwood forest that represented the dominant class of land cover with a largest patch size of 2,657 m^2^ (Table 1). Although sparse, other classes (e.g., pasture) were present and elevation ranged from 406 m to 785 m.

**Table 1:** Forest type, physiographic province^a^, and proportions of 3 classes of land cover summarized within the extent of each study area used in analysis of home range for white-tailed deer (*Odocoileus virginianus*) from 2009 to 2015 in Pennsylvania, USA.

Forest type	Physiographic province	Developed	Forested	Open
Appalachian oak	Gettysburg-Newark Lowland	0.162 (62)	0.221 (84)	0.617 (639)
Appalachian oak	Pittsburgh Low Plateau	0.100 (33)	0.623 (390)	0.277 (125)
Northern hardwoods	Glaciated Low Plateau	0.037 (17)	0.717 (929)	0.246 (143)
Appalachian oak	Appalachian Mountain	0.064 (27)	0.723 (660)	0.213 (252)
Appalachian oak	Appalachian Mountain	0.073 (21)	0.676 (779)	0.251 (366)
Northern hardwoods	Deep Valleys	0.018 (15)	0.893 (2657)	0.089 (110)

The average land cover patch area is in parenthesis next to each land cover type.

^a^Bureau of Topographic and Geologic Survey, Commonwealth of Pennsylvania Department of Conservation and Natural Resources.

## Results

**Figure 3:**
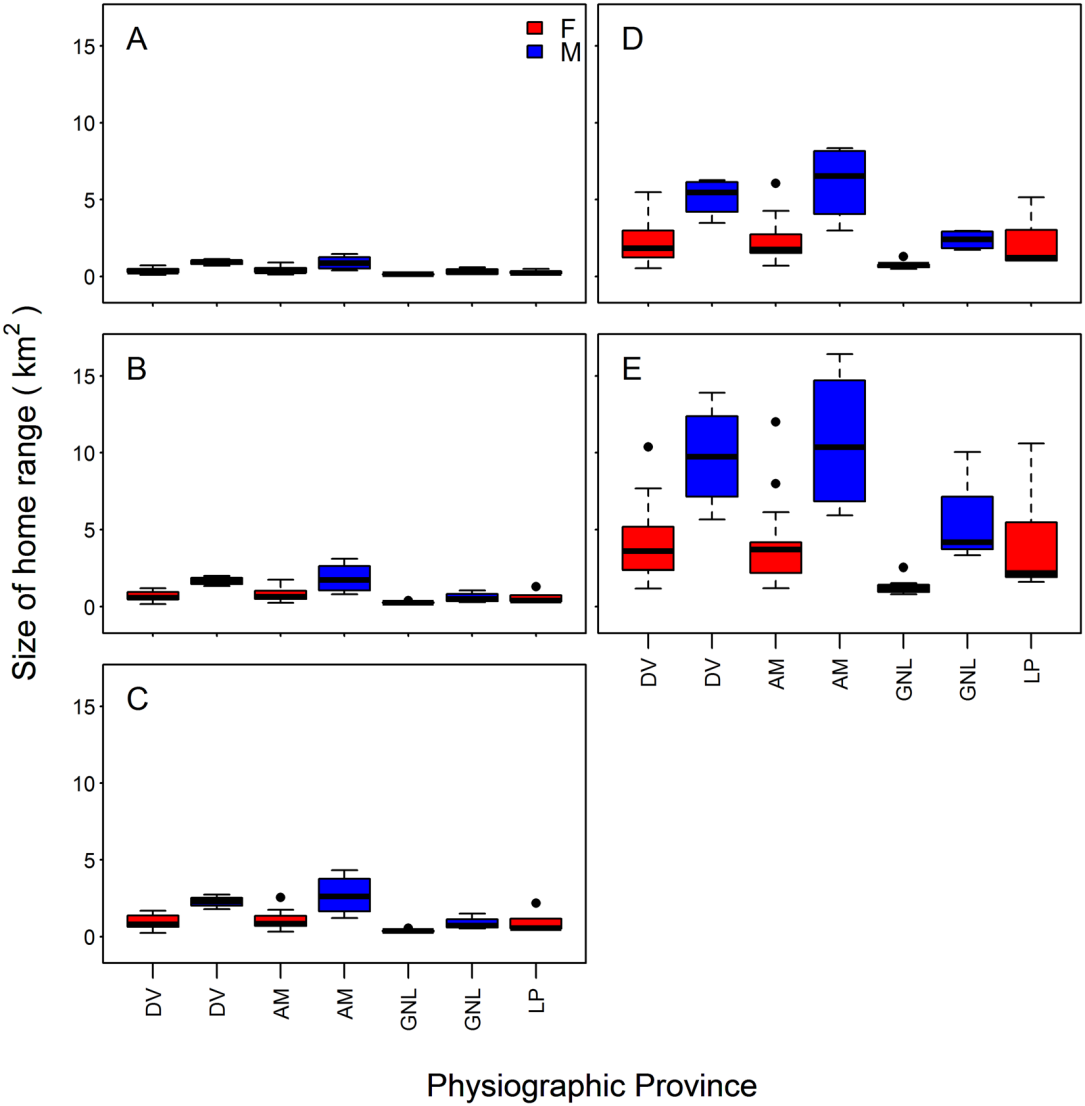
Boxplot of mean size of home range (km^2^) by sex and habitat heterogeneity for white-tailed deer (*Odocoileus virginianus*) from 2009 to 2015 in Pennsylvania, USA for (**A**) 50%, (**B**) 70%, (**C**) 80%, (**D**) 95%, and (**E**) 99% isopleths representing spatial scales of study. Mean size (±SD) was by physiographic provinces: Gettysburg-Newark Lowland (GNL), Pittsburgh and Glaciated Low Plateau (LP), Appalachian Mountain (AM), and Deep Valley (DV). Generated with Rstudio (version 1.0.153, www.rstudio.com).

Size of 95% home range for females ranged from 0.50 km^2^ in the Gettysburg-Newark Lowland to 5.49 km^2^ in the Deep Valleys (Fig. 3). Size of 95% home range for males ranged from 1.73 km^2^ in the Gettysburg-Newark Lowland to 8.36 km^2^ in the Low Plateau regions (Fig. 3). Males established mean 95% home ranges (4.55 km^2^) that were more than twice the size of mean home ranges established by females (1.92 km^2^) across all study areas (*F*_1,54 = _32.15, *P* < 0.001). We observed no sex-area interaction (*P* = 0.939) but we observed differences in mean 95% home range in Gettysburg-Newark Lowland (1.26 km^2^) and Deep Valleys (2.79 km^2^; *P* < 0.001; Fig. 3). Mean size of 99% home range also varied between males (8.66 km^2^) and females (3.63 km2; *F*_1,54_ = 30.12, *P* < 0.001). We observed no sex-area interaction (*P* = 0.599) but we observed differences in mean 99% home range in Gettysburg-Newark Lowland (2.57 km2) and Deep Valleys (5.30 km2; *P* < 0.001).

**Figure 4:**
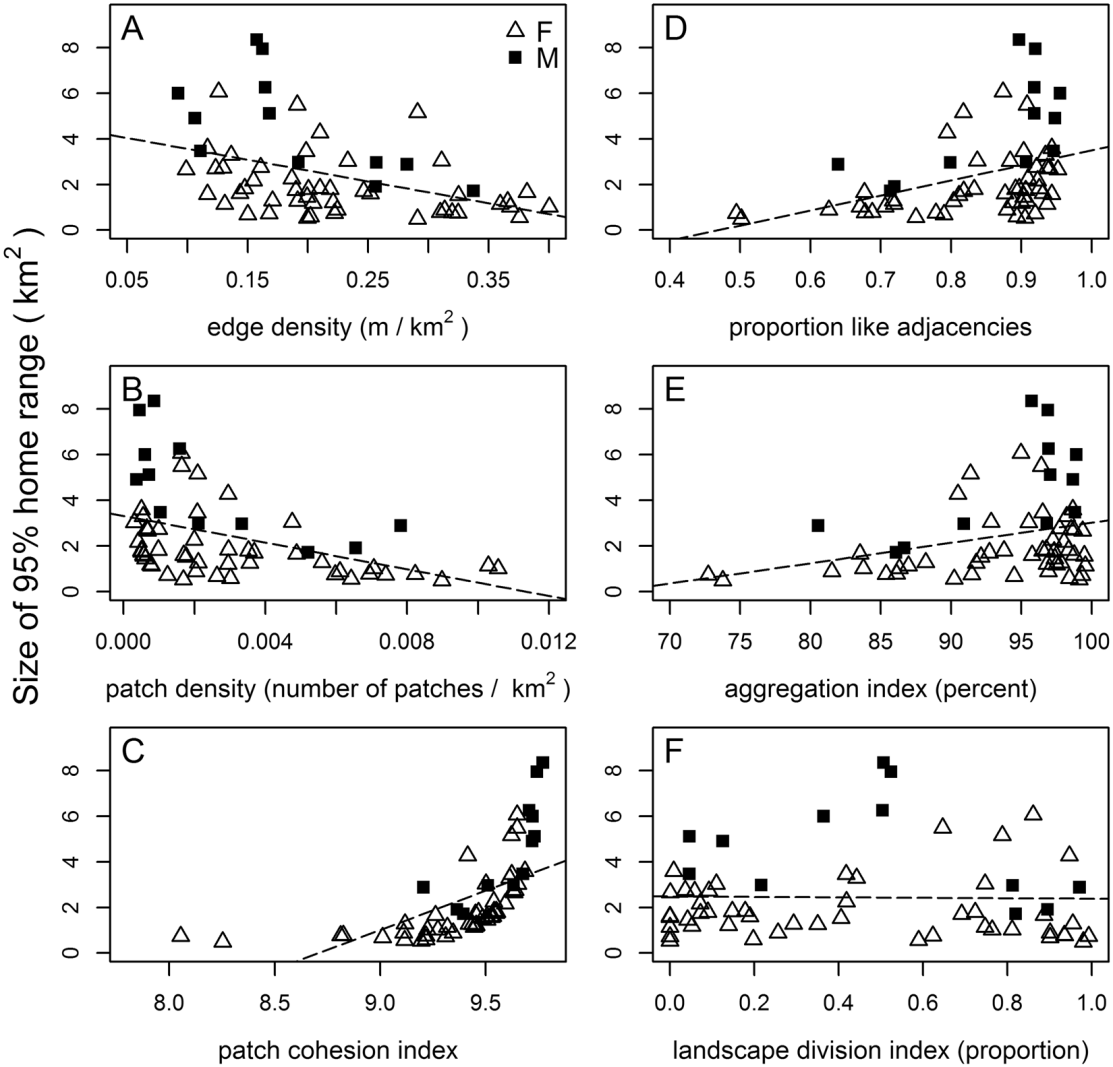
Relationship between size of 95% home range (km^2^) by sex of white-tailed deer (*Odocoileus virginianus*) from 2009 to 2015 in Pennsylvania, USA and various area, edge and aggregation metrics: (**A**) edge density, (**B**) patch density, (**C**) patch cohesion index, (**D**) proportion like adjacencies, (**E**) aggregation index, and (**F**) landscape division index. Generated with Rstudio (version 1.0.153, www.rstudio.com).

Increased levels of edge density and patch density were indicative of more fragmented landscapes across our study areas and corresponded with a decrease in size of home range (Fig. 4A,B). Patch cohesion index, proportion of like adjacencies, and aggregation index were high across our study sites indicating considerable connectivity of homogeneous forested areas. An increase in these three aforementioned metrics corresponded with an increase in size of home range (Fig. 4C–E). Landscape division index varied across our study areas with values closer to zero reflecting landscapes consisting of a single patch compared to values closer to one indicating a decrease in forest and forest patch size. Size of home range exhibited no relationship with landscape division index across our study areas suggesting this metric may not be suitable for describing size of home range (Fig. 4F).

The model containing patch cohesion index was the most supported model of our landscape metrics and size of home range at all spatial scales. This model accounted for all the AIC_c_ weights regardless of spatial scale of analysis with no other models within 2.0 ΔAIC from the top model. Parameter estimates were 0.677 and 2.028 at our smallest (50% isopleth) and largest (99% isopleth) scales, respectively. Patch cohesion index was positively related to size of home range (Fig. 4C).

## Discussion

Although our samples size was limited for males, we expected size of home range to vary between sexes, given associations of larger home range with males, as well as increased movements by males for dispersal, migration, and breeding^25–28^. Larger home range for males in our study area was consistent with findings in similar studies on white-tailed deer that identified several factors that influenced differences in size between sexes, including increased nocturnal movement by males during the breeding season (e.g., October–November), decreased diurnal movement by females during the growing season (e.g., June–July), and higher site fidelity by females across all seasons^26,29,30^. Increased rates and distances of dispersal were observed for males in the Northeast and Midwest^27,28^ and supported our findings of larger home range and greater daily distance traveled by males than females in each study area, regardless of habitat configuration, in which both sexes were included for analysis^8^.

Home ranges for males and females were smaller in the more fragmented landscapes (e.g. high edge density, low proportion like adjacencies) than home ranges in all other study areas. Dechen-Quinn *et al*.^19^ observed smaller home ranges in more fragmented areas for female white-tailed deer. It is possible that home ranges that were smaller in more fragmented areas of our study were related to the landscape comprised of small woodlots and high intensities of open and developed classes that may provide forage for white-tailed deer^11,19,31^. One of our study areas more closely resembled an agricultural landscape than any other study area, and size of annual home range for females in this area (0.63 km^2^) were comparable to mean annual home ranges for female white-tailed deer in agricultural landscapes of the Midwest (0.99–1.47 km^2^)^15^ but less than in an agricultural-forested landscape in the Northeast (2.03 km^2^; Fig. 2)^19^. In areas where open and developed classes were as prominent as they were in Gettysburg-Newark Lowland, deer likely were able to obtain suitable forage without being required to traverse large distances^19,32^.

Deer located in Deep Valleys study area established home ranges that were largest of all deer in our analysis. Areas that were predominantly forested may be viewed as less productive, because deer that rely on food sources (e.g., mast and browse) that can vary on a seasonal or annual basis may be required to establish home ranges that are larger to ensure access to sufficient resources^10,33,34^. In areas of Virginia that were predominantly forested, mast can comprise >76% of the diet of deer during fall and winter months^35^. Female deer in Virginia also expanded seasonal home ranges into oak (*Quercus* spp.) stands during years in which production of mast exceeded 300 kg/ha, whereas size of home range remained unchanged during years of poor mast production (<100 kg/ha)^33^. Production in these areas of Virginia varied greatly from year to year (e.g., 396 kg/ha to 3 kg/ha) and suggested that shifts in home range by deer in predominantly forested landscapes, that are similar to our study regions, are in response to availability of forage.

In some study areas, changing the spatial scale from 50% to 99% encompassed greater amounts of edge and other patches that were occupied less often by deer. Conversely, changing the scale in areas with larger forested patches encompassed only greater amounts of forest rather than other classes of land cover in the homogeneously forested landscape in the Deep Valleys. Our model selection results, however, remained the same regardless of the scale that was used. These findings were inconsistent with findings in female mule deer in the West and white-tailed deer in the Northeast, where measures of landscape that influenced size of home range changed as the scale of analysis was increased from 250 m to 5,000 m from the centroid location of each home range^30,36^. In a similar study of female white-tailed deer in the Midwest, measures of landscape that influenced size of home range changed as the spatial scale was modified^15^. Although changing spatial scale yielded similar results in our study, we are the first to report on spatial scales as determined by home ranges estimated using MKDE with GPS data in North America. The inherent nature of MKDE as a more refined estimator of home range (i.e., contours that follow the shape of non-stationary location data) compared to traditional KDE^1^ requires further evaluation to determine if our selection of estimator influenced similarities in spatial scale and if this would be similar for other cervid species.

A caveat of our study was that we were unable to assess deer densities across our study areas. High deer densities in Gettysburg-Newark Lowland (>40 deer/km^2^)^37^ were linked to crop damage^11^, and similar densities (47–51 deer/km^2^) were identified as the cause of forest regeneration issues on a predominantly forested landscape in Connecticut^38^. Deer in high-density areas also have been shown to exhibit greater site fidelity, especially during winter months^39^. Reduction in deer density appears to have varying effects on expansion of home range. Seasonal home range expanded by 30% in a developed area in South Carolina following a 50% reduction in herd size^40^, however, home ranges remained unchanged in Connecticut following reduction of the deer herd from 88 to 17 deer/km^2^)^41^. Although assessments of home range expansion in response to herd reduction were not feasible for our study, lower densities (~18 deer/km^2^)^42^ in our Deep Valleys area identified by the largest tracts of homogeneous forest (Table 1) may require males to traverse greater distances in search of females during the breeding season. Therefore, landscape is one of many factors that likely play a role in differences in size of home range between more fragmented and homogeneously forested landscapes.

Relatedly, spread of disease likely is related to the scale at which deer establish home range, and our findings show that this scale (i.e., size of home range) varies considerably between more fragmented versus homogeneously forested landscapes. Disease surveillance efforts in more fragmented areas could be concentrated locally to reflect concentrated movements and higher contact rates between social groups^43,44^, or conversely, surveillance in homogeneously forested areas could be conducted at a broader scale to reflect home ranges that are more expansive and dispersed due to limitations in foraging and breeding opportunities. Regardless of impetus for research, variability in size of home range, dispersal, and movements have been documented between fragmented, heterogeneous landscape in comparison to more homogenous landscapes for a variety of cervids^16,36,45^. Although methods to determine spatial scale varies considerably depending on objectives or researcher preferences, relationships of smaller home ranges within more heterogeneous and fragmented landscapes appears consistent across cervid species. Further evaluation of methodologies to measure metrics for consistency across studies and species at the landscape-scale (e.g., buffered circles) and local scale (e.g., home ranges isopleths) appears warranted.

## Methods

### Size of Home range

We captured and equipped 61 white-tailed deer with GPS collars across the study areas for various projects on white-tailed deer movements and survival between 2009 and 2015^17,37,46^. All GPS collars had accuracy <10 m and fix success rates of >96% based on manufacturer recommendations (Supplemental Table 1). We captured deer using a combination of rocket nets^47^, single-gate Clover traps^48^, and drop nets (modified from^49^). Only adult deer (>2 years of age) were included in our analysis because dispersal occurs between 1 and 2 years of age in both sexes^17,50^ and these populations lack migratory movements to winter deer yards or similar habitat shifts^19^. All capture and handling methods were in accordance with protocols approved by the Pennsylvania State University Institutional Animal Care and Use Committee (IACUC No. 29677 and 34910) and within guidelines of the American Society of Mammalogists^51^. We estimated mean home range for each deer using the *adehabitatHR* package in program R (^52,53^ R Foundation for Statistical Computing, Vienna, Austria).

We incorporated duration of time between recorded locations, a minimum distance of 30 m that needed to be traveled between consecutive locations to be considered active, and landscape-specific diffusion coefficients into MKDE to estimate 50%, 70%, 80%, 95%, and 99% isopleths of annual home range for each deer (Supplemental Table 2). We defined annual as late winter of one year through late winter of the following year with no overlapping dates (e.g., 1 February–31 January) because dates of capture varied for each deer. We grouped deer according to sex and physiographic province and used a two-way ANOVA with a confidence level of 95% (α = 0.05) to assess differences in size of home range between sexes and landscape with post-hoc differences determined by Tukey’s multiple comparison test.

### Land cover reclassification

We reclassified the 2011 National Land Cover Database with 30 m resolution into 5 classes: developed, forested, open, water, and wetland^54^. Prominent water sources and wetlands were present in only 2 study areas and represented less than 1% of the landscape in home ranges of 5 deer in these areas. Therefore we did not consider these 2 classes in our analysis and reclassified water as open (e.g., pastures, grasslands and croplands) and wetland as forested, given the association of woody wetlands with forest vegetation. The developed class contained roads and all intensities of development, including urban, suburban, and exurban. We extracted the forested class from the data for analysis because forested habitat would be the most influential given the association of forest with movement and disease epidemiology in white-tailed deer in the Northeast and Midwest^28,50,55^.

### Landscape metrics

We calculated metrics of landscape configuration and connectivity for the forested class of land cover for each deer within 5 isopleths or spatial scales (50%, 70%, 80%, 95% and 99% isopleths of home range) using the SDMTools package in program R. We calculated 6 metrics for forest class of land cover across a variety of landscapes that reflected edges and aggregation of like habitats known to influence size and shape of home ranges based on previous research linking landscape heterogeneity to size of home range for white-tailed deer, red deer (*Cervus elaphus*) and roe deer (*Capreolus capreolus*)^15,16,19^. Our analysis included *edge density* (m/ha) because white-tailed deer are known to respond to edge habitats. Our analysis also included five aggregation metrics: *patch density* (number of patches/km^2^), *patch cohesion index* (measure of physical connectivity of forest), *proportion of like adjacencies* (measures the degree of aggregation of patch types accounting for patch size and shape), *aggregation index* (area-weighted mean class aggregation index), and *landscape division index* (probability that two randomly chosen pixels are not in the same patch). We selected these covariates due to their relationships with definitions of spatial heterogeneity and landscape configuration and connectivity^56,57^. We also limited our analysis to aggregation metrics of only the forest class because white-tailed deer are known to respond to amount and configuration of forested landscapes in the eastern and Midwestern U.S. and because we were comparing landscapes with varying extents of configuration and connectivity^15,19,50^.

### Statistical analysis

We created 7 linear models *a priori* with the six covariates as independent variables and natural log of home range as the response variable for each spatial scale along with an intercept only model. We used covariates that corresponded to each spatial scale to determine if changing the scale of analysis from 50% to 99% would influence model selection results. We selected home range contours^15,19^ over buffered circles due to the arbitrary nature of defining radii of buffered circles in previous research^30,36,45^. We used Akaike’s Information Criterion with correction for small sample size to evaluate the set of models at each spatial scale (AIC_c_)^58^.

## Electronic supplementary material

Supplementary Information
