# Supplementary material for: Heterogeneity of a landscape influences size of home range in a North American cervid
Source: Sci Rep. 2018 Oct 2;8:14667. doi: 10.1038/s41598-018-32937-7 (PMC6168582; doi:10.1038/s41598-018-32937-7)
Supplement: Supplementary file 1 — Supplementary Information [file 41598_2018_32937_MOESM1_ESM.docx]

**Heterogeneity of a landscape influences size of home range in a North American cervid**

W. David Walter^1,†^, Tyler S. Evans,^2,4^ David Stainbrook,^2,5^ Bret D. Wallingford,^3^ Christopher S. Rosenberry,^3^ Duane R. Diefenbach^1^

^1^U.S. Geological Survey, Pennsylvania Cooperative Fish and Wildlife Research Unit, The Pennsylvania State University, University Park, PA 16802, USA

^2^Pennsylvania Cooperative Fish and Wildlife Research Unit, The Pennsylvania State University, University Park, PA 16802, USA

^3^Pennsylvania Game Commission, Bureau of Wildlife Management, Harrisburg, PA 17110, USA

^4^Present address: West Virginia Division of Natural Resources, French Creek, WV 26218, USA

^5^Present address: Massachusetts Division of Fisheries and Wildlife, Westborough, MA 01581, USA

*^†^****E-mail:*** *wdw12@psu.edu*

Supplemental Table 1. A summary of capture methods, radiocollars, and radiotelemetry sampling designs for the physiographic province^a^ included in landscape-level assessment of female white-tailed deer home ranges in Pennsylvania, 2011−2015.

| **Variables** | **Gettysburg-Newark Lowland** | **Pittsburgh and Glaciated Low Plateau** | **Appalachian Mountain** | **Deep Valleys** |
| --- | --- | --- | --- | --- |
|  |  |  |  |  |
| Capture method^b^ | RN (S), CT | DN (S), RN (S), CT | DN (S), RN (S), CT | DN (S), RN (S), CT |
| Sample Size by sex | 4 (M), 9 (F) | 6 (F) | 4 (M), 18 (F) | 4 (M), 16 (F) |
| Collection schedule (hours) | 5 mins Aug, Nov, Jan, Apr; 1 hour remainder | 24 hours 16 Jul–30 Apr; 1.5 hours 1 May–15 Jul | 7 hours 1 Jan–30 Sep; 3 hours 1 Oct – 27 Nov; 20 mins 28 Nov–31 Dec | 7 hours 1 Jan–30 Sep; 3 hours 1 Oct – 27 Nov; 20 mins 28 Nov–31 Dec |
| Manufacturer^c^ | VEC | VEC, TEL, HAB | VEC, TEL, HAB | VEC |
| Number of Locations | 44,527 | 21,926 | 81,219 | 78,322 |
| Citation | Stainbrook 2011 | Lutz et al. 2015 | Lutz et al. 2015 | On-going |

^a^ Bureau of Topographic and Geologic Survey, Commonwealth of Pennsylvania Department of Conservation and Natural Resources.

^b^ DN = drop net, RN = rocket net, CT = single-gate Clover traps; S in parenthesis refers to method used along with sedation using 0.5 mg/kg body mass of xylazine hydrochloride

^b^ TEL = Telonics, Inc., Mesa, AZ, USA; VEC = Vectronic Aerospace GmgH, Berlin, Germany; HAB = H.A.B.I.T. Research Ltd., Victoria, British Columbia

**Literature Cited**

Lutz, C. L., D. R. Diefenbach, and C. S. Rosenberry. 2015. Population density influences dispersal in female white-tailed deer. Journal of Mammalogy 96:494-501.

Stainbrook, D. P. 2011. Methods of estimating white-tailed deer abundance at Gettysburg National Military Park: testing assumptions of distance sampling. The Pennsylvania State University, University Park, PA.

Table 2. Number of locations and size of 50%, 70%, 80%, 95% and 99% home range (km^2^) for female (F) and male (M) white-tailed deer (*Odocoileus virginianus*) between 2009 and 2015 in Pennsylvania, USA.

| Study area | Sex | Locations | 50% | 70% | 80% | 95% | 99% |
| --- | --- | --- | --- | --- | --- | --- | --- |
|  |  |  |  |  |  |  |  |
| Low Plateau | F | 4063 | 0.2009 | 0.3784 | 0.5477 | 1.2591 | 2.1727 |
| Low Plateau | F | 4032 | 0.2246 | 0.4067 | 0.5443 | 1.0249 | 1.6172 |
| Low Plateau | F | 3583 | 0.1799 | 0.3199 | 0.4608 | 1.0246 | 1.9274 |
| Low Plateau | F | 3261 | 0.2072 | 0.3650 | 0.5260 | 1.1426 | 2.1658 |
| Low Plateau | F | 3713 | 0.4946 | 1.3047 | 2.1799 | 5.1597 | 10.6135 |
| Low Plateau | F | 3274 | 0.3248 | 0.7395 | 1.1640 | 3.0334 | 5.4662 |
| Gettsyburg-Newark Lowland | M | 4878 | 0.1302 | 0.2961 | 0.5296 | 1.7257 | 3.3360 |
| Gettsyburg-Newark Lowland | F | 3148 | 0.0930 | 0.1786 | 0.2489 | 0.4952 | 0.8019 |
| Gettsyburg-Newark Lowland | F | 3156 | 0.1907 | 0.3126 | 0.4118 | 0.8841 | 1.5314 |
| Gettsyburg-Newark Lowland | F | 3044 | 0.16722 | 0.2962 | 0.3984 | 0.7355 | 1.1231 |
| Gettsyburg-Newark Lowland | M | 3085 | 0.5932 | 1.0644 | 1.4960 | 2.9625 | 4.2358 |
| Gettsyburg-Newark Lowland | F | 3044 | 0.0993 | 0.1807 | 0.2551 | 0.5596 | 0.9489 |
| Gettsyburg-Newark Lowland | F | 2872 | 0.1499 | 0.2991 | 0.4137 | 0.7777 | 1.2193 |
| Gettsyburg-Newark Lowland | F | 2995 | 0.1345 | 0.2533 | 0.3629 | 0.7501 | 1.1859 |
| Gettsyburg-Newark Lowland | M | 4451 | 0.3284 | 0.5776 | 0.7727 | 1.9187 | 4.1169 |
| Gettsyburg-Newark Lowland | F | 2985 | 0.1232 | 0.2508 | 0.3621 | 0.7639 | 1.4242 |
| Gettsyburg-Newark Lowland | F | 4495 | 0.1373 | 0.2487 | 0.3314 | 0.5979 | 0.8502 |
| Gettsyburg-Newark Lowland | M | 4816 | 0.2023 | 0.4061 | 0.6735 | 2.8835 | 10.0540 |
| Gettsyburg-Newark Lowland | F | 1558 | 0.1877 | 0.3879 | 0.5575 | 1.2838 | 2.5502 |
| Appalachian Mountain | F | 3412 | 0.2950 | 0.5388 | 0.7285 | 1.7871 | 6.1222 |
| Appalachian Mountain | F | 2328 | 0.2315 | 0.4396 | 0.6137 | 1.2218 | 1.9505 |
| Appalachian Mountain | F | 2925 | 0.1277 | 0.2532 | 0.3767 | 0.8840 | 1.5842 |
| Appalachian Mountain | F | 2600 | 0.1447 | 0.2485 | 0.3355 | 0.6865 | 1.2118 |
| Appalachian Mountain | F | 3724 | 0.5521 | 1.1536 | 1.7416 | 4.2663 | 7.9863 |
| Appalachian Mountain | F | 3689 | 0.4420 | 0.8780 | 1.1815 | 2.5206 | 5.6924 |
| Appalachian Mountain | F | 2955 | 0.8928 | 1.7585 | 2.5503 | 6.0593 | 12.0128 |
| Appalachian Mountain | M | 2673 | 1.4555 | 3.0951 | 4.3391 | 8.3573 | 12.9993 |
| Appalachian Mountain | F | 2934 | 0.3250 | 0.6011 | 0.8387 | 1.8006 | 3.8649 |
| Appalachian Mountain | F | 1748 | 0.2697 | 0.4946 | 0.7013 | 1.5291 | 2.3586 |
| Appalachian Mountain | F | 2712 | 0.3099 | 0.6468 | 0.9065 | 1.6004 | 2.1997 |
| Appalachian Mountain | F | 2893 | 0.6794 | 1.0827 | 1.3563 | 2.1702 | 3.1943 |
| Appalachian Mountain | F | 2887 | 0.4784 | 0.7861 | 1.0069 | 1.7498 | 3.2471 |
| Appalachian Mountain | M | 2915 | 1.0678 | 2.1574 | 3.1849 | 7.9552 | 16.4176 |
| Appalachian Mountain | F | 2872 | 0.7209 | 1.2134 | 1.5970 | 3.0275 | 5.4480 |
| Appalachian Mountain | M | 2700 | 0.3954 | 0.8039 | 1.2222 | 2.9819 | 5.9268 |
| Appalachian Mountain | F | 2718 | 0.5324 | 1.0428 | 1.4407 | 2.7305 | 4.1713 |
| Appalachian Mountain | F | 2415 | 0.4208 | 0.6550 | 0.8473 | 1.4590 | 2.0474 |
| Appalachian Mountain | F | 2735 | 0.2690 | 0.5032 | 0.6910 | 1.6603 | 3.6926 |
| Appalachian Mountain | F | 2698 | 0.5587 | 0.9799 | 1.3430 | 2.7507 | 4.1706 |
| Appalachian Mountain | F | 2724 | 0.2292 | 0.4575 | 0.6750 | 1.7100 | 4.1596 |
| Appalachian Mountain | M | 2937 | 0.6422 | 1.3139 | 2.0646 | 5.1149 | 7.7331 |
| Deep Valleys | F | 2707 | 0.1214 | 0.2789 | 0.4636 | 1.2796 | 2.0362 |
| Deep Valleys | M | 2478 | 0.9871 | 1.7477 | 2.3442 | 4.9146 | 8.6153 |
| Deep Valleys | M | 2922 | 0.7031 | 1.3404 | 1.8013 | 3.4741 | 5.6641 |
| Deep Valleys | F | 2694 | 0.6043 | 1.0386 | 1.4135 | 2.7034 | 3.8362 |
| Deep Valleys | F | 2731 | 0.2940 | 0.6046 | 0.9893 | 3.4561 | 7.6742 |
| Deep Valleys | F | 2941 | 0.5247 | 0.9358 | 1.3281 | 3.2843 | 7.2109 |
| Deep Valleys | F | 2890 | 0.7126 | 1.1948 | 1.5323 | 2.6524 | 4.2320 |
| Deep Valleys | F | 2936 | 0.4919 | 0.8112 | 1.0526 | 1.8286 | 2.9704 |
| Deep Valleys | F | 2690 | 0.2751 | 0.5272 | 0.7585 | 1.6179 | 2.9565 |
| Deep Valleys | F | 2721 | 0.4729 | 1.0515 | 1.6159 | 3.5932 | 5.6023 |
| Deep Valleys | M | 2701 | 1.1232 | 1.9979 | 2.7369 | 5.9996 | 13.9122 |
| Deep Valleys | F | 1664 | 0.0929 | 0.1705 | 0.2382 | 0.5303 | 1.1774 |
| Deep Valleys | F | 1885 | 0.1468 | 0.2626 | 0.3593 | 0.7274 | 1.3924 |
| Deep Valleys | F | 2742 | 0.3484 | 0.6292 | 0.8367 | 1.5739 | 3.3610 |
| Deep Valleys | F | 2890 | 0.2472 | 0.4914 | 0.7342 | 2.2562 | 4.7432 |
| Deep Valleys | M | 2707 | 0.8890 | 1.5804 | 2.2475 | 6.2600 | 10.8498 |
| Deep Valleys | F | 2689 | 0.3074 | 0.5581 | 0.7437 | 1.8340 | 4.6992 |
| Deep Valleys | F | 1916 | 0.2799 | 0.4743 | 0.6338 | 1.1838 | 2.3334 |
| Deep Valleys | F | 2551 | 0.2767 | 0.4889 | 0.6493 | 1.1425 | 2.4354 |
| Deep Valleys | F | 2607 | 0.4474 | 0.9650 | 1.6963 | 5.4893 | 10.3734 |
